# Trait ontology analysis based on association mapping studies bridges the gap between crop genomics and Phenomics

**DOI:** 10.1186/s12864-019-5812-0

**Published:** 2019-06-03

**Authors:** Qingchun Pan, Junfeng Wei, Feng Guo, Suiyong Huang, Yong Gong, Hao Liu, Jianxiao Liu, Lin Li

**Affiliations:** National Key Laboratory of Crop Genetic Improvement, Huazhong Agricultural University, Wuhan, 430070 China

**Keywords:** Trait ontology, Functional genomics, Enrichment analysis, Maize, Rice

## Abstract

**Background:**

Trait ontology (TO) analysis is a powerful system for functional annotation and enrichment analysis of genes. However, given the complexity of the molecular mechanisms underlying phenomes, only a few hundred gene-to-TO relationships in plants have been elucidated to date, limiting the pace of research in this “big data” era.

****Results**:**

Here, we curated all the available trait associated sites (TAS) information from 79 association mapping studies of maize (*Zea mays* L.) and rice (*Oryza sativa* L.) lines with diverse genetic backgrounds and built a large-scale TAS-derived TO system for functional annotation of genes in various crops. Our TO system contains information for up to 18,042 genes (6345 in maize at the 25 k level and 11,697 in rice at the 50 k level), including gene-to-TO relationships, which covers over one fifth of the annotated gene sets for maize and rice. A comparison of Gene Ontology (GO) vs. TO analysis demonstrated that the TAS-derived TO system is an efficient alternative tool for gene functional annotation and enrichment analysis. We therefore combined information from the TO, GO, metabolic pathway, and co-expression network databases and constructed the TAS system, which is publicly available at http://tas.hzau.edu.cn. TAS provides a user-friendly interface for functional annotation of genes, enrichment analysis, genome-wide extraction of trait-associated genes, and crosschecking of different functional annotation databases.

****Conclusions**:**

TAS bridges the gap between genomic and phenomic information in crops. This easy-to-use tool will be useful for geneticists, biologists, and breeders in the agricultural community, as it facilitates the dissection of molecular mechanisms conferring agronomic traits in an easy, genome-wide manner.

**Electronic supplementary material:**

The online version of this article (10.1186/s12864-019-5812-0) contains supplementary material, which is available to authorized users.

## Background

Due to the overwhelming success of high-throughput molecular techniques such as microarray analysis and next-generation sequencing, increasing numbers of genes are continuously being identified and studied, increasing the need for functional annotation. Gene ontology (GO) is a biological classification system that employs a common vocabulary of gene and protein functions across species [1]. The GO system was constructed based on the assumption that a large fraction of the genes specifying core biological functions are shared by all eukaryotes [1]. GO provides multifaceted functional descriptions of biological processes, molecular functions, and cellular components for a large quantity of genes [2]. GO analysis has become the most widely used system for functional annotation of genes. Many GO databases have been created for animals and plants that are primarily based on orthologous relationships with genes in the GO databases for yeast, *Drosophila melanogaster* (fruit fly), and mice [2]. For example, AgriGO is an outstanding GO toolkit that is widely used by the agronomic community [3–5].

Because GO terms were established based on analysis of core biochemical pathways and do not illustrate regulatory relationships, the GO terms utilized in plants are sometimes confusing and ambiguous, especially for plant geneticists, biologists, and breeders. Therefore, many new ontological classification systems have been developed. MetaCyc, a metabolic pathway database, was constructed to illustrate relationships among genes in various pathways [6]. Such metabolic pathway databases have been used to annotate microbial genomes and have been expanded for use in higher plants [7–9]. In addition, Plant Ontology (PO), a hierarchical ontology, was designed to specifically describe plant growth, developmental stages, and plant morphology [10]. PO uses the same data model as GO but contains more phenotypic information [10–17]. Like PO, Trait Ontology (TO) was developed based on the morphological characteristics of different organisms using a consistent vocabulary [18–21].

For both animals and plants, TO annotations utilize the Entity-Quality (EQ) model, which ensures consistency across different species [22–26]. Oellrich focused on mutant phenotypes associated with genes of known sequence in *Arabidopsis*, maize, *Medicago*, rice, soybean, and tomato to construct a shared TO dataset, which could be used for cross-species querying and semantic similarity analyses [27]. TO is the most comprehensive system available to date that annotates the traits of various plant species using a single, universal vocabulary [27]. However, due to its complexity, only a fraction of genes has thus far been annotated and assigned TO terms, which has hampered the use of this system.

Association mapping, including genome-wide association analysis (GWAS) and candidate gene resequencing followed by association mapping, is a reliable method for ascertaining the statistical relationships between genes and phenotypes [28, 29]. Over the past 20 years, association mapping has matured rapidly and has been used to identify tens of thousands of gene-to-trait relationships in plants [30–32]. By combining large-scale phenotyping of natural populations with information from high-density markers and sophisticated statistical genetic models, GWAS and candidate gene association mapping have proven to be powerful methods for identifying candidate causal genes [32]. The integration of association mapping results from different studies, including analyses of plants with different genetic backgrounds, could provide ample evidence for gene-to-trait relationships at the genome-wide scale [33, 34]. Therefore, the development and rapid progress of association analysis in plants have made it an unprecedented resource for constructing TO systems.

With the rapid progress in techniques for high-throughput mRNA sequencing, a large set of plant transcriptome profiles can now be obtained [35–39]. Increasing numbers of co-expression networks are being constructed and have proven to be conserved, which indicates biological significance [40, 41]. Co-expression networks are emerging as efficient tools for deciphering the potential functional roles of genes along with the GO, TO, and PlantCyc metabolic pathway systems. The relationships between the GO, co-expression networks, PlantCyc metabolic, and TO databases can be described as follows: the GO and PlantCyc metabolic databases contain the most basic functional and regulatory information about genes. Co-expression networks extend this information to describe the complex roles of genes at the transcriptomic level based on GO and PlantCyc analyses. TO contains the most phenotypic information among the databases. Based on the relationships between the four tools, the potential molecular mechanisms of genes affecting a specific phenotype can be uncovered.

Integrating the diverse information obtained from GO, TO, PlantCyc, and co-expression networks would provide a unique opportunity to decipher multiple functional aspects of a gene of interest. The Gramene database contains combined, multifaceted biological data, including genomic, transcriptomic, proteomic, phenomic, and metabolic information across tens of plant species, providing a comprehensive bioinformatics platform in plants [18, 19]. However, to perform functional annotation and enrichment analysis of genes, public research information must be accurately curated, organized, and integrated in terms of GO, TO, PlantCyc pathways, and co-expression networks. In the current study, we collected information from as many association mapping studies as possible in maize and rice, curated the gene-to-TO relationships based on the association mapping results, and constructed a large-scale TO database across different linkage disequilibrium decay (LD) distances. A comparison of TO vs. GO enrichment analysis showed that Trait Associated Site (TAS)-derived TO represents a powerful alternative tool for functional annotation and enrichment analysis. Our comprehensive functional annotation and enrichment platform, which is based on the integration of the TO, GO, PlantCyc, and co-expression networks databases, bridges the gap between genomic and phenomic information in crops.

## Results

### A comprehensive trait ontology (TO) system in crops

**Fig. 1:**
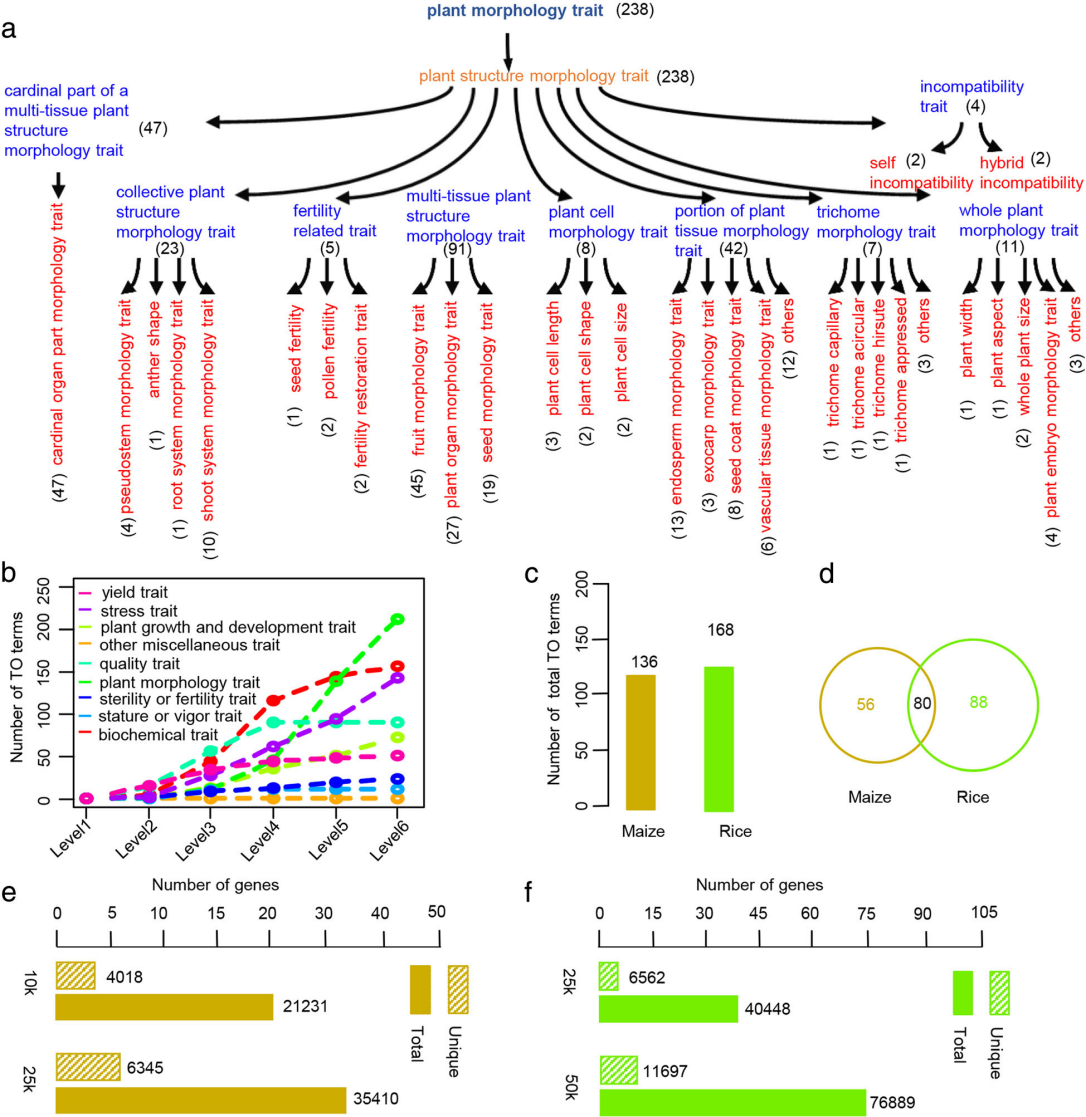
Large-scale TAS-derived TO database derived from association mapping studies in maize and rice. **a** Hierarchical structure of TO terms. Number in parentheses represents the number of derived TO terms. **b** Number of TO terms in each TO category. **c** Number of TO terms classified in maize and rice. **d** Overlap of TO terms between maize and rice. (**e**) and (**f**) Number of gene and gene-to-TO relationships identified at different LD distances in maize and rice, respectively

Trait ontology (TO, Fig. 1a) analysis is an efficient method for investigating the relationships between genes and traits. TO classifies plant traits into nine trait groups, including yield, stress, sterility or fertility, stature or vigor, quality, plant morphology, plant growth and development, biochemical, and other miscellaneous traits and organizes them into hierarchical layers from top (level 1) to bottom (level 6) (Fig. 1b; Additional file 1). To date, 864 TO terms have been defined in plants [15, 18–21]. To construct a genome-wide TO database, we collected the results of 79 association-mapping studies in rice and maize, providing genetic evidence for the relationships between genomes and phenomes in crops (Additional file 2). Curation of these association mapping results showed that 136 and 168 TO terms have been defined in maize and rice, respectively (Fig. 1c). Of the 136 maize TO terms, over half were detected in rice, even though maize and rice differ in morphology (Fig. 1d).

According to the association-mapping model, different linkage disequilibrium (LD) decay distances correspond to different mapping resolutions, suggesting that different gene-to-TO relationships would be obtained at different LD cutoffs. In maize, 4018 unique genes were found to be associated with the 136 TO terms, which represent 21,231 gene-to-TO relationships across different association-mapping studies, assuming an LD distance of 10 kb (Fig. 1e). The number of maize genes reached 6345, corresponding to 35,410 gene-to-TO relationships, given an LD distance of 25 kb. Since the LD in the rice association-mapping panel decays over much longer distances [32], the LD cutoff used for the construction of the TO database in rice was higher. A total of 6562 rice genes were associated with the 168 TO terms under an LD cutoff of 25 kb, which represents 40,448 gene-to-TO relationships (Fig. 1f). However, the gene-to-TO number reached 76,889 at an LD cutoff of 50 kb. Overall, we curated over 100,000 gene-to-TO relationships in maize and rice (Additional files 3 and 4), representing the largest TO system available for crops.

### Comparison of TO vs. GO shows that TAS-derived TO is an effective alternative tool for functional annotation and enrichment analysis of genes

**Fig. 2:**
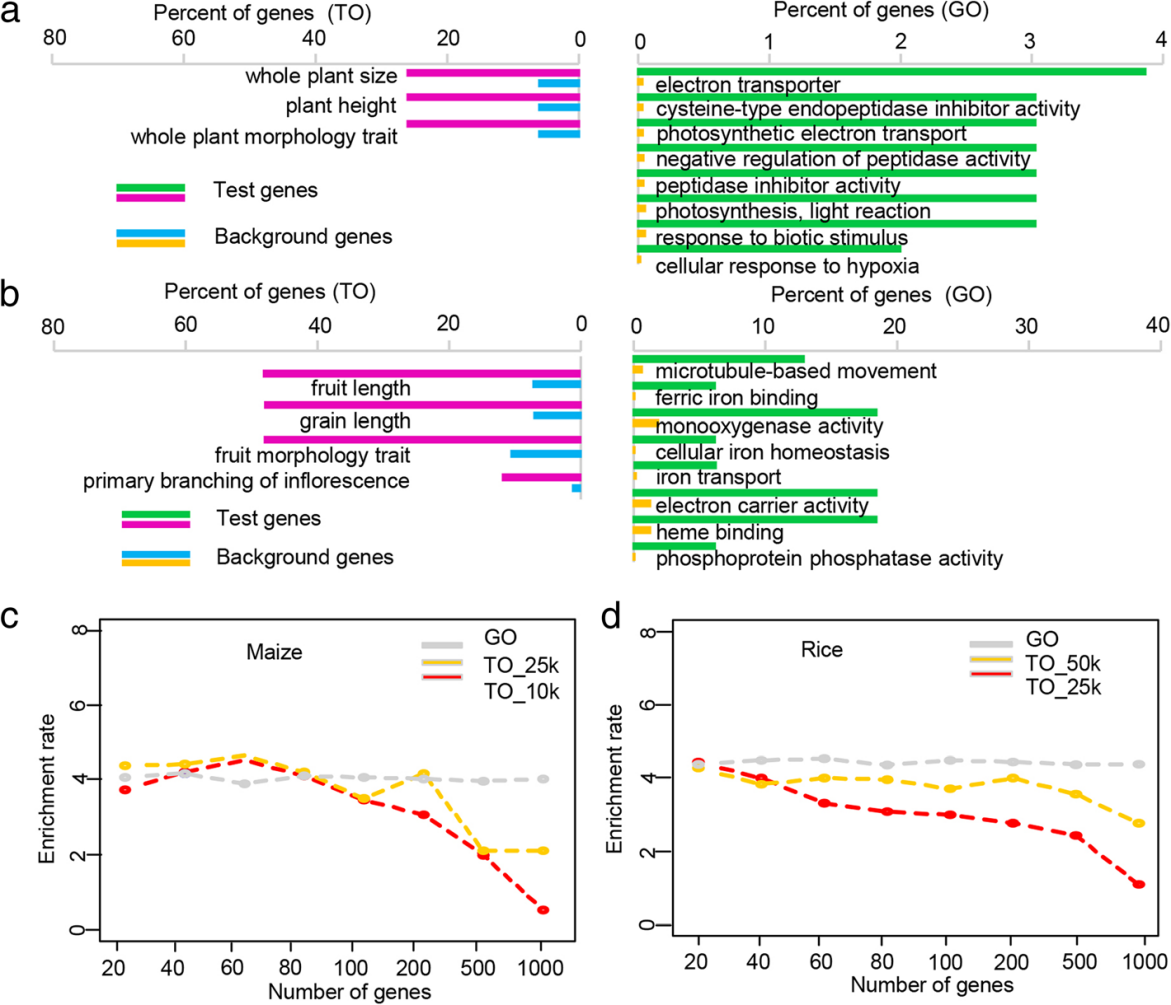
Comparisons of TO and GO analyses indicating that TAS-derived TO is an efficient alternative tool for functional analysis of genes. **a b** Bar charts of over-represented TO terms (left) and GO terms (right) for 145 plant height-related DEGs in maize (**a**) and kernel size-related genes in rice (**b**). The X-axis shows the percentage of genes and represents the abundance of the TO and GO terms. The percentage for the tested gene list was calculated as the number of genes mapped to TO or GO terms divided by the total number of all genes in the test list. The same calculation was employed for the background gene list. The Y-axis shows different TO and GO terms. (C) (D) False-positive rates of TO and GO enrichments in maize (**c**) and rice (**d**). The X-axis shows the number of randomly selected genes for the test, and the Y-axis shows the false-positive rate

We previously conducted RNA-seq of the top-most leaves of near isogenic maize lines carrying a recessive mutation in a plant height gene and their wild-type counterpart and identified 146 differentially expressed genes (DEGs) [42]. These DEGs are thought to be involved in plant development. To test the robustness of TO, we used these DEGs as input for TO and GO enrichment analyses. A substantial number (30/146) of DEGs were related to 42 TO terms, while 103 were associated with 258 GO terms (Additional file 5). Of these functional terms, three TO categories were significantly enriched among these DEGs, and a higher number of GO terms were enriched (Fig. 2a; *P* < 0.01). Additionally, major functional roles (stress response and plant growth) were consistently identified in both analyses. However, TO provided more specific functional annotation than GO. For example, TO analysis specifically indicated that plant height and whole plant morphology traits were associated with these 146 input DEGs, whereas GO analysis only provided some conceptual functional annotations, such as cell tip growth, photosynthesis, and light reaction, although the GO annotations made biological sense (Additional file 5).

We also used 20 well-known rice genes reported to control kernel size to test the robustness of our TAS-derived TO system for estimating the rate of false negatives [43]. Six of the 20 well-known rice kernel size genes were associated with 23 TO terms, and a comparable proportion of these genes were related to 34 GO terms (Additional file 6). As expected, these genes were significantly enriched in TO terms including grain length, fruit length, fruit morphology trait, and other traits related to kernel size (Fig. 2b; *P* < 0.01). On the other hand, conventional GO analysis of these genes showed significant enrichment for GO terms microtubule-based movement, phosphoprotein phosphatase activity, and others; these results make some biological sense but are not sufficiently specific. Analyses of both maize and rice indicated that TAS-derived TO enrichment has a false negative rate comparable to that of GO analysis but provides more detailed and intuitive functional annotation information.

To test the false-positive rate (FPR) of TAS-derived TO, we conducted 1000 simulations of randomly selected gene sets of variable size via TO and GO enrichment analyses. In maize, the average FPR of 1000 simulations of randomly selected gene sets via GO analysis was approximately 4% and remained stable across different gene numbers. The average FPR for TAS-derived TO in maize was close to that of GO when the gene numbers were 20, 40, 60, or 80 but dropped off rapidly, to close to 1%, when the gene number increased to 1000 (Fig. 2c). Interestingly, except for the simulations using 20 genes, in which the FPRs for TO and GO were similar, the FPRs for all simulations in rice were significantly lower for TO than for GO (Fig. 2d). These findings suggest that TO enrichment has a comparable or lower FPR than GO. Therefore, our TAS-derived TO system represents a powerful tool for functional annotation and enrichment analysis of genes in maize and rice.

### TAS-derived TO is an excellent tool for the systematic dissection of molecular mechanisms underlying agronomic traits

**Fig. 3:**
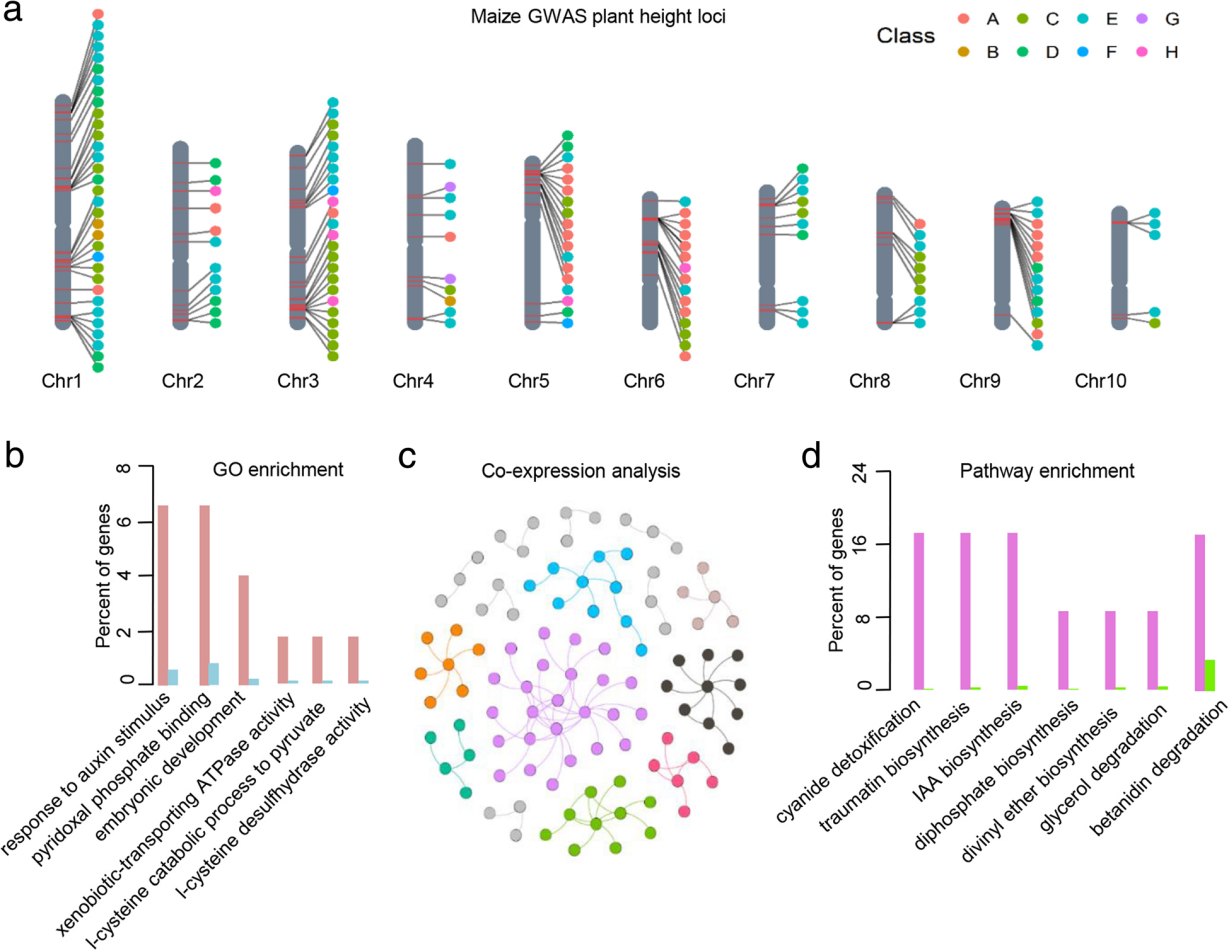
A case study of the analysis of plant height using our TO system. **a** Genome-wide distribution of plant-height-associated genes in maize. Each dot represents one plant-height-associated gene. Different colors indicate different association mapping studies. **b** Bar chart of overrepresented GO terms for plant-height-associated genes. The X-axis shows different GO terms, and the Y-axis shows the percentage of genes with annotated GO terms. **c** Co-expression relationships of plant-height-associated genes. Different colors represent different co-expression module. **d** Bar chart of over-represented PlantCyc metabolic pathways for plant-height-associated genes. The X-axis and Y-axis are as described for Fig. 3b

We collected and curated all results of association mapping studies of phenotypic variation in maize and rice using the TO scheme, providing an unprecedented opportunity to systematically dissect the possible molecular mechanisms associated with various agronomic traits. Taking plant height as an example, we integrated information for 625 functional genes and found that they were associated with plant height variation in maize (Additional file 7). Based on the top 10% of the genes most significantly associated with this trait from each association mapping study, we identified 135 functional genes, which are distributed across all 10 maize chromosomes (Fig. 3a). GO enrichment analysis showed that the plant height-related genes were associated with the categories ‘response to stimulus’ and ‘organelle organization’ (Fig. 3b). Transcriptome profiling indicated that most of these plant height-related genes are located in the same co-expression network (Fig. 3c). Plant metabolic pathway analysis also indicated that most plant height-related genes are involved in IAA biosynthesis, xylose degradation, and other metabolic pathways (Fig. 3d). As expected, 18% of these top 10% of plant height-related genes encode enzymes in the IAA biosynthesis pathway, which is in agreement with previous results (Fig. 3d) [44]. It is worth noting that 18% of plant-height-associated genes are involved in betanidin degradation, providing new targets for research focusing on plant height. Similarly, genes from the divinyl ether biosynthesis, glycerol degradation, diphosphate biosynthesis, and cyanide detoxification pathways were significantly enriched among our TO plant height terms, suggesting that highly complex molecular mechanisms underlie plant height variation in crops. Together, these results suggest that these genes might function in a concerted manner at both the biochemical and transcriptomic levels. Using our TAS-derived TO database, it is easy to extract all candidate functional genes associated with a specific trait, providing a genome-wide overview of the molecular mechanisms underlying plant traits.

**Fig. 4:**
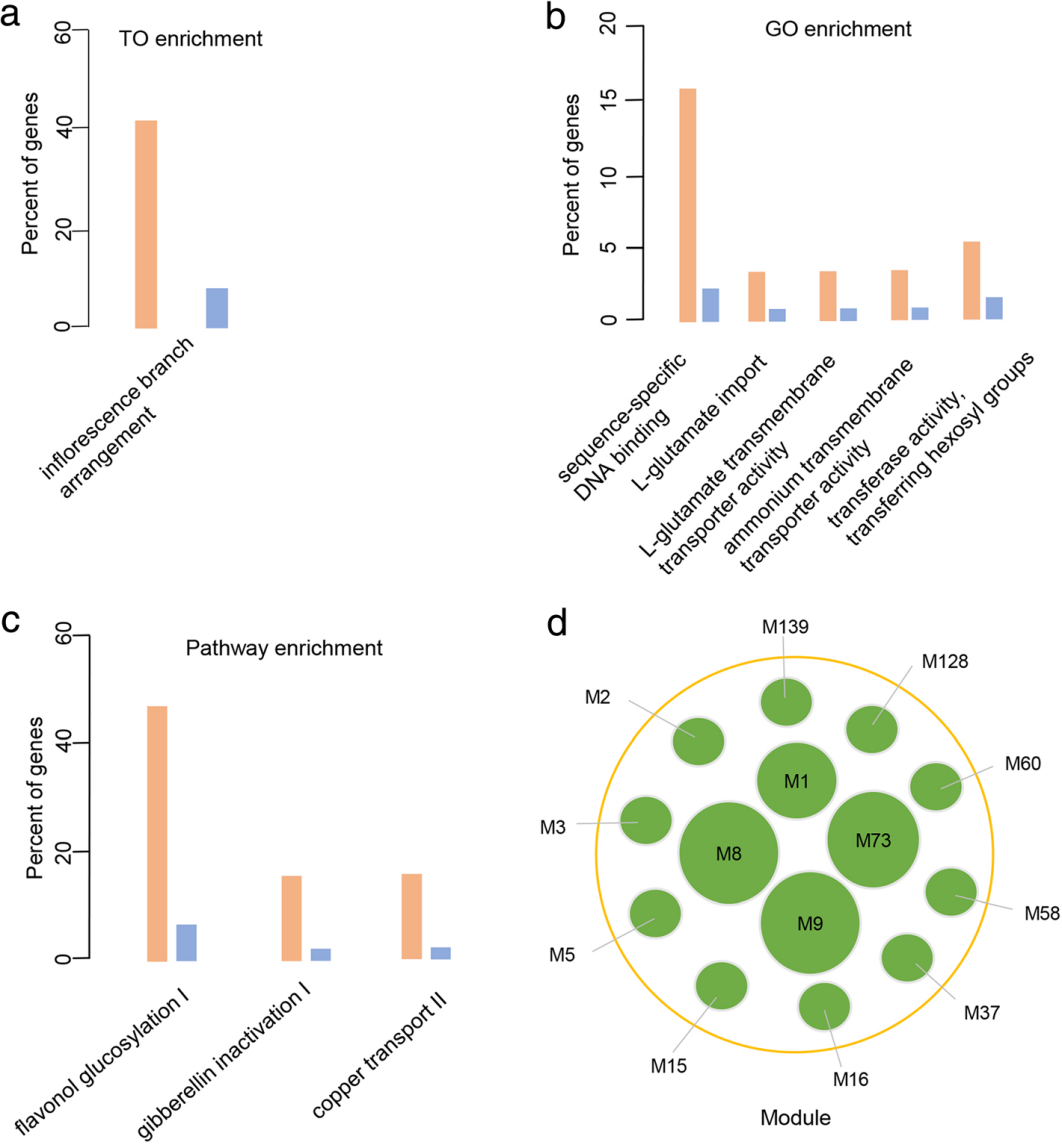
A case study of maize internode-specific gene annotation using TO. (**a**)-(**c**) Functional enrichment of internode cell-specific genes in the TAS-derived TO, GO, and PlantCyc metabolic pathway databases, respectively. Brown represents input and blue represents the reference control. **d** Co-expression modules containing maize internode-specific genes. Each green circle represents one co-expression module. The bigger the circle, the greater the number of internode-specific genes in a specific module

The TAS-derived TO system is also a good resource for dissecting the molecular mechanisms underlying plant development. Here, we used laser microdissection RNA-seq data from 2-week-old maize B73 seedlings to determine the transcriptome of internode cells, which contributes to the development of axillary meristems and plant height [45, 46]. Shannon entropy analysis across different tissues/stages uncovered 44 internode-specific genes [41, 47]^.^ TO analysis using these 44 genes showed that they are enriched in the TO term ‘inflorescence branch arrangement’, as expected (Fig. 4a) [45, 46]. These 44 internode-specific genes were enriched in similar functional categories in the GO and plant metabolic pathway databases, which is suggestive of functional identity (Fig. 4b; 4c). Moreover, co-expression network analysis indicated that some of these internode-specific genes were co-expressed within four co-expression modules and shared co-expression relationships among different modules, which is suggestive of potential coordinated transcriptome patterns for these internode-specific genes (Fig. 4d). Together, these results indicate that our TAS-derived TO system provides biologically meaningful insights into gene function and cellular or developmental processes.

### TAS is an integrative toolkit for functional annotation and enrichment analysis of genes in crop species

**Fig. 5:**
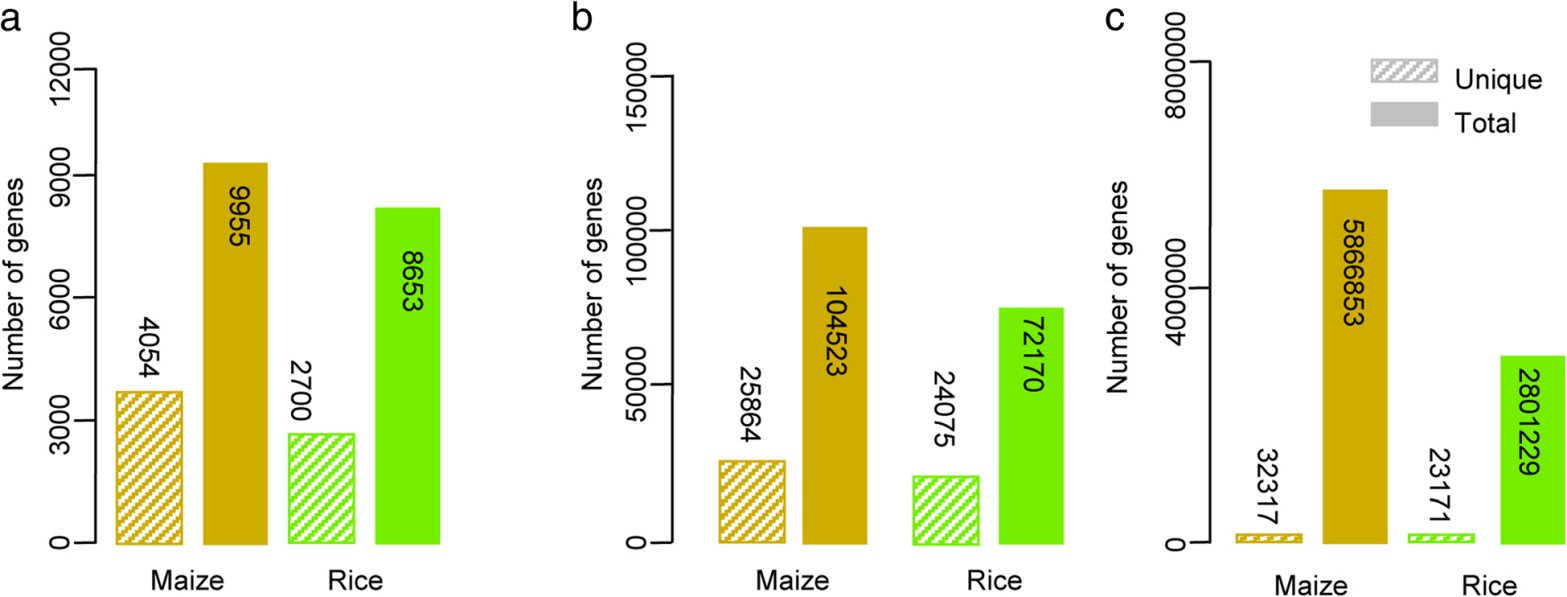
Number of genes from the PlantCyc, GO, and co-expression databases integrated in TAS. (**a**)-(**c**) Number of unique genes and their relationships from the PlantCyc, GO, and co-expression databases, respectively

**Fig. 6:**
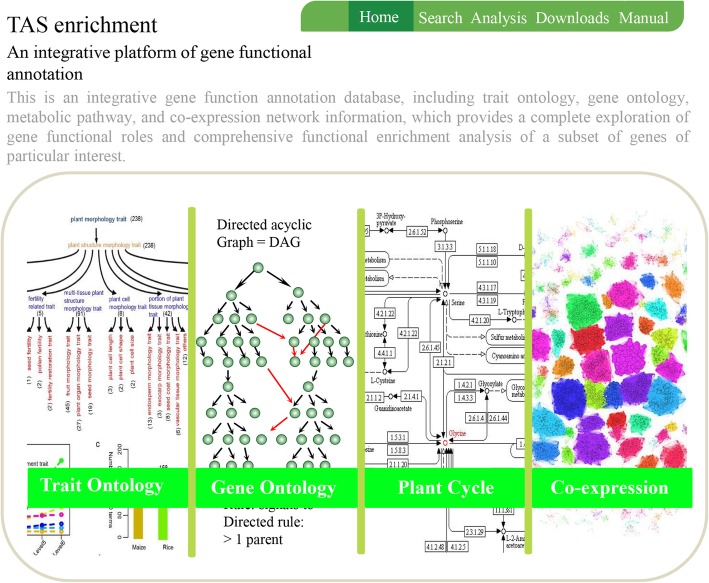
The user-friendly TAS interface. Through this interface, one can submit a search query of a subset of genes, and the TAS system can extract all of the TAS-derived TO, GO, PC, and co-expression information and perform enrichment analyses

To provide a systematic gene annotation and enrichment analysis platform, we integrated the curated TAS-derived TO database with the PlantCyc database, GO database, and co-expression network data and constructed TAS, an integrative toolkit for functional genomics in maize and rice (http://tas.hzau.edu.cn/). In addition to information for thousands of genes and tens of thousands of gene-to-TO relationships, TAS contains information for 4054 genes in 422 metabolic pathways and 2700 genes in 336 metabolic pathways in maize and rice, respectively (Fig. 5a). Over 24,000 genes in both maize and rice have GO annotations and were integrated into the TAS database (Fig. 5b). TAS also lists over 32,000 maize genes that are co-expressed in 189 co-expression modules and 23,171 co-expressed rice genes in 187 co-expression modules, both of which were constructed based on transcriptome profiling across different tissues/stages of reference inbred lines or cultivars (Fig. 5c). TAS has a user-friendly interface that plant geneticists, biologists, and breeders can use to search and annotate the functional roles of query genes (Fig. 6).

**Fig. 7:**
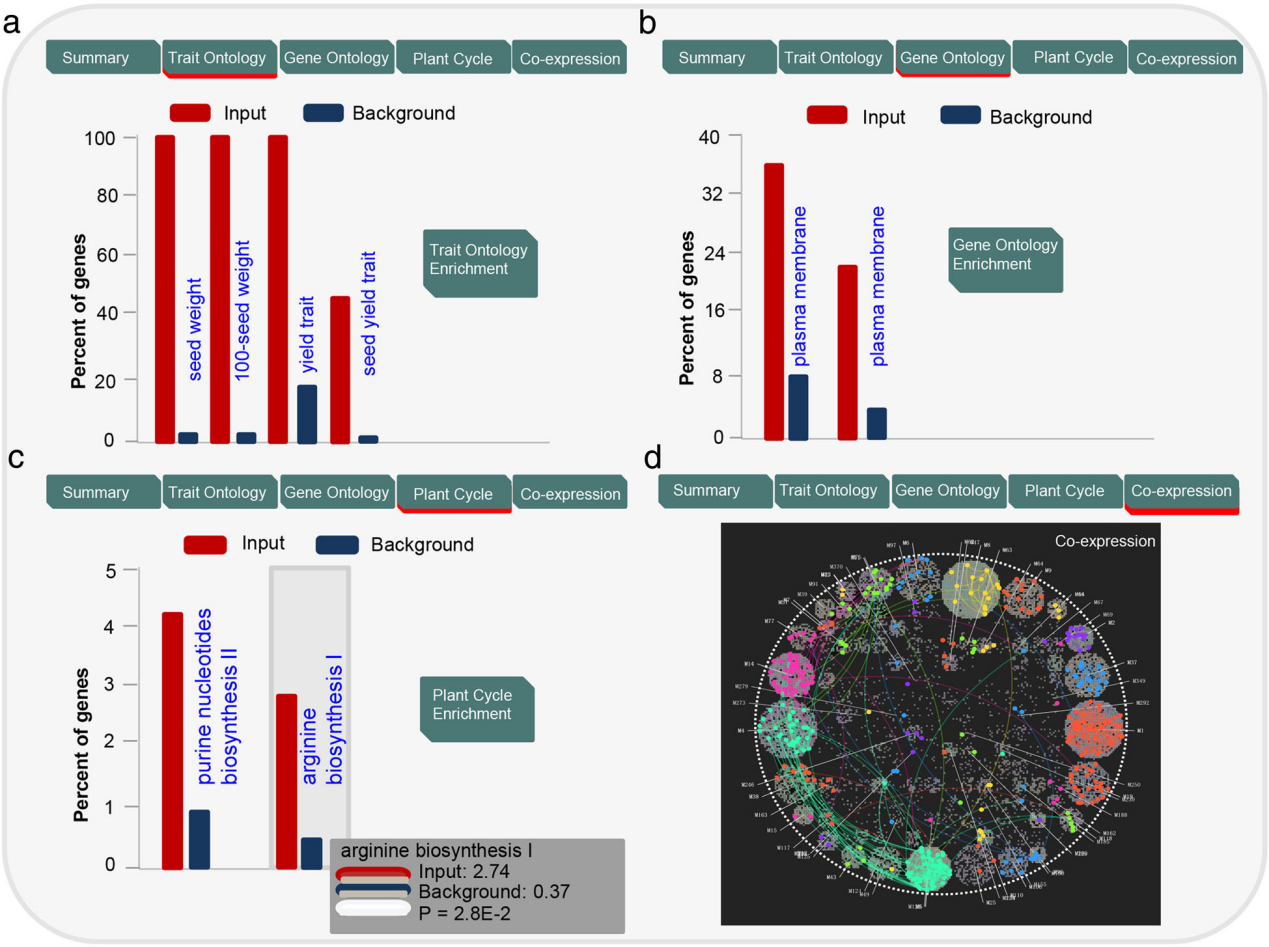
An example of enrichment analysis in TAS. (**a**)-(**d**) Examples of enrichment analysis in TAS for TO, GO, PC, and co-expression networks, respectively. **a** Results of TO enrichment analysis of query genes associated with grain yield in maize (performed with our TAS system using trait search item “Kernel Rows in Number”). **b** Results of GO enrichment analysis of query genes associated with grain yield in maize (using trait search item “Kernel Rows in Number”). **c** Results of PlantCyc enrichment analysis using query genes associated with plant architecture in maize (using trait search item “Plant height”). **d** Co-expression network of genes associated with plant height in maize (using trait search item “Plant height”)

Importantly, the TAS platform also provides a comprehensive analysis toolkit, enabling enrichment analysis and cross-comparison across TO, GO, PlantCyc, and co-expression network results (Fig. 7a-d), enhanced graphical presentation of functional annotation and enrichment analysis of genes, and other features, such as downloading, an updating service, and so on. TAS has the following features: 1) a user-friendly data extraction interface, allowing researchers to extract all annotated gene-to-TO, gene-to-GO, and gene-to-PC (PlantCyc) terms using queried genes of interest and to retrieve all related genes for a queried agronomic trait; 2) Tools for enrichment analysis, allowing users to query genes of interest for associations with specific agronomic traits, fundamental biological functions, and biological metabolic pathways. Users can input gene lists into the TAS platform and submit the enrichment analysis query, which will return all-in-one enrichment results for TO, GO, PC, and co-expression network modules; all of these results can be cross-compared; 3) Enhanced graphical presentation of all enrichment results. Researchers can download the entire original TO, GO, PC, and co-expression networks in bulk or retrieve the raw results of each analysis in Excel format for further analysis.

## Discussion

In this era of big data, our understanding of the functional roles of genes lags far behind the generation of high-throughput sequencing and phenomics data. The hierarchical design of the ontology of the GO, PO, and TO databases provides an efficient way to assess the multifaceted functional roles of genes of interest. In this study, we integrated all publicly available association mapping data from maize and rice and built a large-scale TO database for crops. Furthermore, we created an integrative bioinformatics platform that combines GO, TO, PlantCyc (metabolic pathway), and co-expression network information, enabling comprehensive functional annotation and enrichment analysis. This platform provides a user-friendly interface for searching and analyzing the functional roles of genes, bridging the gap between genomic and phenomic information in crops.

Most TO terms have been established largely based on association mapping results across different genetic populations. Association mapping has long been used to identify candidate genes and has proven to be an effective way to detect the relationships between genes and traits [32, 48]. However, the results of such analyses frequently lack experimental validation. Notably (except for well-studied model organisms such as *Arabidopsis*, yeast, and fruit fly), GO, PO, and metabolic annotations have mainly been generated by bioinformatic prediction [49]. Given the rapid decay of LD in maize and rice [30, 32], the resolution of association mapping analysis can sometimes reach a single or very few genes, improving the reliability of TO terms. However, in some genomic regions, LD decay occurs at a considerably slower rate, increasing the probability of obtaining multiple false positives. Therefore, it is essential to critically analyze the results with an understanding of the genomic regions under consideration. In addition, the ability to obtain association mapping data from plants with diverse genetic backgrounds and to perform hand-curation of each TO term and cross-checking between TO, GO, metabolic pathway, and co-expression networks provide users with ample information about the likely functions of the genes of interest.

The current TAS-derived TO system covers one-quarter of the annotated gene sets in maize and rice. Since maize and rice are the most important staple food crops in the world, large worldwide research communities have been carrying out numerous association mapping studies of both crops every year since 2010. The TO database is designed to facilitate the continued integration of new association mapping results and can easily be expanded to incorporate information from additional plant species. Since over half of the TO terms are consistent across different species, our TAS platform will add ortholog alignment information to expand the TO terms for each species based on comparative genomics.

Unlike many other bioinformatic platforms, TAS provides an integrative gene functional annotation and enrichment database with data analysis toolkits. With the continuing accumulation of functional gene annotations, TAS will become an increasingly powerful platform for facilitating research into the molecular mechanisms conferring agronomic traits in an easy, genome-wide manner.

## Conclusions

In this big data era, massive amounts of biological data can now be obtained simultaneously. However, it is becoming increasingly challenging to interpret these available data. We devised a new alternative method for annotating gene functions and functional enrichment for a large set of genes by integrating Trait Ontology design with the results of association mapping studies. Comparative analyses showed that our TAS-derived TO system is an effective alternative method compared to GO analysis. By combining TO with association mapping results, we can better understand the relationships between phenomes and genomes in a wide range of species. Our method for functional annotation and enrichment analysis of genes can easily be utilized for other plants, expanding our knowledge of plant species far beyond maize and rice.

## Methods

### Trait ontology annotation and classification

Trait ontology (TO) was devised to further understand the molecular processes occurring in organisms. The phenotypes of six plant species (including maize and rice) were previously compared and unified into a consistent vocabulary of trait descriptions with well-known functionally validated genes, phenotypes, and genomic database information [27]. Here, the *Entity-Quality* (EQ) method was used to assure phenotypic consistency between species. *Entity* refers to organisms, species, and traits, while *Quality* refers to how the trait variation is described, such as big/small, increased/decreased temperature, round, reduced length, and so on. Gene function refers to the annotated gene function. The EQ method can be used to transform these descriptions into numerical values. The following formula serves as an example:

$$ \mathrm{EQ}=\mathrm{Entity}\ \left(\mathrm{Organisms}+\mathrm{Species}+\mathrm{Trait}\ \right)+\mathrm{Quality}\ \left(\mathrm{trait}\ \mathrm{value}\ \mathrm{or}\ \mathrm{character}+\mathrm{gene}\ \mathrm{function}\right) $$

The EQ method is sufficient for defining phenotypes and could be used to improve the consistency of phenotypic descriptions. A correlation matrix was constructed for different species and phenotypes. Based on a similarity matrix, the information content *I* (*t*) of an ontology class *t* was defined based on the probability *P*(*X* = *t*) that a phenotype is characterized by t:

$$ I=-\log \left(P\left(X=t\right)\right) $$

P (X = t) is the phenotypic value, which was calculated for the phenotype matrix. For two phenotypes, P and R, P represents the ontology classes Cl (P) = P1…Pn and R represents the ontology classes Cl (R) = R1…Rm. The similarity patterns of phenotypes P and R could be obtained using the following formula [50]:

$$ sim\ \left(P,R\right)=\frac{\sum_{x\in Cl(R)\cap Cl(P)}I\ \left(\mathrm{x}\right)}{\sum_{y\in Cl(R)\cup Cl(P)}I\ \left(\mathrm{y}\right)} $$

The phenotypes were classified into different trait terms based on the similarity phenotypic values. In total, based on the relationships between genes and plant traits, the consistent trait annotations were divided into different hierarchical groups, which were classified by subset (Additional file 1) [27, 51]. The trait ontology annotation and trait identity (TO: ID) values were collected from the official TO website. Nine primary TO terms and six hierarchical groups of trait annotation and IDs were implemented in our study.

### Data mining of published association mapping studies in maize and rice

Genome-wide association analysis is a method for dissecting the relationships between genes and phenotypic variations in crops. All published association-mapping studies in maize and rice were queried by searching NCBI PubMed using the key words “*maize*”, “*rice*”, and “*association mapping*”. The 30 different types of association results from the literature were sorted, including the trait name, physical marker loci, population size, population type, minor-allele frequency, marker size, physical version, reference name, significant *p*-value, nearest gene name, gene function annotation, gene GO information, final physical locations of markers (maize for V3 and rice for *MSU*7.0), mutation location information, TO term anchored information, and so on. The method used to obtain the TO term anchored information is described in the next section. The mutation location was extracted using “snpEff” software. The final physical locations of markers were obtained using BLAST software. The functional annotations of genes were obtained using InterProScan, and the gene GO information was downloaded from the agriGO V2 website [5]. The remaining functional annotation information was obtained using Perl and R scripts.

### Construction of TAS-derived trait ontology terms in crops

The traits described in the literature were organized into consistent trait tables based on semantic similarity, trait descriptions in the literature, and trait annotation tables. The maize and rice traits described in the literature are listed in Additional file 2. Finally, genes with significant association mapping signals were assigned to different TO terms (Additional files 3 and 4). Different species have variable LD distances when *r*^*2*^ equals 0.1 across different population sizes and types. For maize, significant association signals within the flanking 10 kb (assuming LD =10 kb) and 25 kb (assuming LD =25 kb) regions of genes were extracted for the identification of trait-associated genes. Two different TAS groups of genes were constructed based on their different levels of LD. Rice has a relatively slow LD rate. Therefore, two different TAS groups were constructed for LD of 25 kb and LD of 50 kb in rice.

### Trait ontology enrichment analysis

A hypergeometric test was employed for TAS-derived TO enrichment analysis in both maize and rice. The input file was the table of gene associations at 10 kb and 25 kb for maize and 25 k and 50 k for rice provided on our website. Briefly, assuming a list of “P” genes tested for maize and rice, given that “M” input genes exist in the database, for a certain TO:ID, there are “k” genes that are associated and “M-k” genes that are not associated. The database has a total number of N genes, and only n genes were associated with this TO:ID. Whether the input genes were associated with this TO term could be calculated using a hypergeometric test, following Fisher’s exact test probability formula:

$$ \mathrm{p}\left(X=\mathrm{k}\right)=\frac{\left(\begin{array}{c}M\\ {}k\end{array}\right)\left(\begin{array}{c}N-M\\ {}n-k\end{array}\right)}{\left(\begin{array}{c}N\\ {}n\end{array}\right)} $$

The value of p was determined using the formula:

$$ \mathrm{p}=1-\sum \limits_{\mathrm{i}=0}^{\mathrm{k}-1}\frac{\left(\begin{array}{c}M\\ {}i\end{array}\right)\left(\begin{array}{c}N-M\\ {}n-i\end{array}\right)}{\left(\begin{array}{c}N\\ {}n\end{array}\right)} $$

Terms with a *p*-value < 0.05 were defined as being enriched.

### Gene ontology enrichment analysis

Gene and GO term information was downloaded from the agriGO website [5]. GO and gene information for maize was downloaded from *Zea mays* locus ID v3.30 (Gramene Release 50), and that for rice was downloaded from *MSU*7.0 gene ID (TIGR; The Institute of Genomic Research Database-TDB). The method for the GO enrichment hypergeometric test was similar to that described above (Trait Ontology enrichment analysis).

### PlantCyc enrichment analysis

The maize and rice Cyc data were downloaded from the PlantCyc website using MaizeCyc version 2.2 and RiceCyc version 3.3. Both gene and pathway information was extracted. The Maize and RiceCyc enrichment analyses were performed using the formula described for TO enrichment analysis.

### Co-expression analysis

Maize and rice gene co-expression data were obtained from a previous study [41]. For maize, RNA-seq data for 64 different tissues/stages were used to profile gene expression values based on the maize reference genome V3 [52]. The expression levels were normalized prior to the construction of co-expression networks for maize and rice [41]. For any pair of genes, the correlation coefficient was calculated using the formula:

$$ r=\frac{\sum \limits_{\mathrm{i}=1}^N\left({x}_{\mathrm{i}}-\overline{x}\right)\left({y}_{\mathrm{i}}-\overline{y}\right)}{\sqrt{\sum \limits_{\mathrm{i}=1}^N{\left({x}_i-\overline{x}\right)}^2}\sqrt{\sum \limits_{\mathrm{i}=1}^N{\left({y}_i-\overline{y}\right)}^2}} $$

The correlation values were then transformed via Fisher transformation [53]:

$$ Z=\frac{1}{2}\mathit{\ln}\frac{1+\mathrm{r}}{1-\mathrm{r}} $$

Finally, the mcl Markov cluster algorithm was used to divide each co-expression network into cluster modules. The formula was as follows:

$$ {\left({\Gamma}_rM\right)}_{pq}=\frac{{\left({\mathrm{M}}_{\mathrm{pq}}\right)}^{\mathrm{r}}}{\sum \limits_{i=1}^k{\left({M}_{iq}\right)}^r} $$

, where r represents the power coefficient [54], k is the gene number, p is the column number of the M matrix, and q is the row number of the M matrix. A default *r* value of 1 was used, and a power coefficient > 1 was used to define the modules. For rice, RNA-seq data from 45 different tissues/stages were used to profile the gene expression values based on genomic information in MSU7.0 [55]. A similar method was used to construct the rice co-expression network.

### False negative and false positive tests of our TAS-derived TO system

To test the robustness of our TAS-derived TO system, two sets of DEGs were used: genes specifically expressed in 2-week-old internode cells, and DEGs between a near-isogenic line (NIL) of a plant height QTL and its wild-type counterpart. RNA-seq data were available for plants in the DE3 and BY815 backgrounds [42]. Second, well-known yield-related rice genes were used as queries [43]. Additionally, GO and TO enrichment results were tested using different numbers of randomly selected genes: 10 kb and 25 kb intervals were tested for maize, and 25 kb and 50 kb intervals were tested for rice. A number of randomly selected sets of 20, 40, 60, 80, 100, 200, 500, and 1000 genes were used as input for the GO and TO enrichment tests. The significant GO and TO numbers, total GO and TO total number, and rates of significant GO and TO term enrichment were summarized and compared.

## Additional files

Additional file 1: **Table S1.** Relationship of different level traits. Level1~7 represent different TO layers from top to bottom. (XLSX 48 kb)

Additional file 2: **Table S2.**Reference information of GWAS. (XLSX 13 kb)

Additional file 3: **Table S3.** 10 k and 25 k TO datasets in maize. (XLSX 1535 kb)

Additional file 4: **Table S4.** 25 k and 50 k TO datasets in rice. (XLSX 3149 kb)

Additional file 5: **Table S5.** Enrichment of TO and GO with DEGs of plant height in maize. The column of “queryitem” represents the number of input genes with the same enrichment term, and the column of “querytotal” represents the total number of input genes. The column of “bgitem” indicates the number of background genes with the same enrichment term, and the column of “bgtotal” indicates the total number of background genes. Pvalue represents the significance level of enrichment. (XLSX 27 kb)

Additional file 6: **Table S6.** Enrichment of TO and GO with kernel-size genes in rice. The meaning of each column is consistent to that of Table S5. (XLSX 12 kb)

Additional file 7: **Table S7**. Trait searched by key word “plant height”. The columns of “Marker_name, chr_ref, Marker_location, Pvalue, indel/snp, MAF” show the information of associated marker with the phenotypic variation. Pvalue indicates the significance level of the association, MAF represents the minor allele frequency of the marker. The columns of “Pop_type, Pop_size, Marker_set, and Model” show the detailed information of association mapping study, such as the marker number for GWAS (Marker_set), and the genetic model used for the association (Model). The columns of “genome_version, Final_version, Chr, Position, chr_gene, start, Gene_refGene, New_gene, Annotation” show the genomic location of the associated marker, the nearby gene, and the annotation of functional nearby gene. The columns of “ID_ref, Ref, note, and Ref_name” present the related reference information. (XLSX 128 kb)

## Data Availability

All of the data and code can be downloaded at https://github.com/panqingchun/Trait-Ontology (Public, free resource website).

## Abbreviations

AM: Association Mapping
DEGs: Differentially Expressed Genes
EQ: Entity-Quality
FPR: False-Positive Rate
GO: Gene Ontology
GWAS: Genome-Wide Association Analysis
LD: Linkage Disequilibrium
PO: Plant Ontology
TAS: Trait Associated Site
TO: Trait Ontology
